# Effectiveness of Repetitive Transcranial Magnetic Stimulation in the Treatment of Depression in the Elderly: A Retrospective Natural Analysis

**DOI:** 10.3390/jcm12144748

**Published:** 2023-07-18

**Authors:** Eisa Almheiri, Abdullah Alhelali, Mohamed A. Abdelnaim, Franziska C. Weber, Berthold Langguth, Martin Schecklmann, Tobias Hebel

**Affiliations:** Department of Psychiatry and Psychotherapy at Bezirksklinikum Regensburg (Medbo KU), Faculty of Medicine, University of Regensburg, 93053 Regensburg, Germanyberthold.langguth@medbo.de (B.L.); martin.schecklmann@medbo.de (M.S.); tobias.hebel@medbo.de (T.H.)

**Keywords:** repetitive transcranial magnetic stimulation, geriatric depression, brain stimulation

## Abstract

Introduction: Depression in the elderly is an understudied condition. Psychopharmacological and psychotherapeutic approaches suffer from specific difficulties with this patient group. Brain stimulation techniques such as repetitive transcranial magnetic stimulation (rTMS) offer a therapeutic alternative. rTMS remains understudied in this age group when compared with younger patients. Methods: A cohort of 505 patients with depression was analyzed in retrospect concerning their response to rTMS treatment. A total of 15.5% were 60 years old or older, defined as the elderly group of depressed patients. The majority of these were treated with high-frequency protocols over the left dorsolateral prefrontal cortex (DLPFC). For group comparisons, we used Student t-tests or chi-square-tests, depending on the scales of measurement. As measures for effect size, we used Cohen’s d for the relative and absolute change in the HDRS total score. Results: Groups did not differ significantly with respect to baseline depression severity or treatment parameters. In the group of elderly patients, a higher number of females were present. Groups did not differ significantly with respect to treatment efficacy, as indicated by the absolute and relative changes in the HDRS-21 sum score. Elderly patients tended to take higher numbers of mood stabilizers. Elderly patients showed a significantly superior reduction for the item “appetite” and a superior reduction tending towards significance for the item “work and interests”. Conclusions: Antidepressant rTMS treatment showed comparable efficacy for patients above 60 years to that in younger patients. Differences between the age groups concerning amelioration of distinct HDRS single items deserve further investigation.

## 1. Introduction

Late-life depression, or depression in the elderly, is often defined as the presence of a depressive disorder in patients over the age of 60. It is often accompanied and its treatment complicated by somatic comorbidity, frailty, and cognitive impairment [1]. Despite a high prevalence of the condition, reaching 10–20% in some surveys, it is often under-reported by patients or misinterpreted due to patients complaining mainly about somatic and cognitive symptoms, making early diagnosis challenging [2]. It has been shown that late-life depression is also associated with an increase in the utilization of health care services and resources, leading to a high economic burden on nations, which indicates the importance of enhancing treatment strategies in this population [3]. Given the current demographic shift, the issue of late-life depression will only become more important to practitioners and health care providers over the next few years and decades. There is also evidence that early- and late-life depression are different in their symptoms, with the elderly showing more somatic symptoms and depression in the elderly being accompanied by cognitive dysfunctions, somatic comorbidity, and physical disability [4]. Inflammatory processes are also discussed [5].

The main treatment modalities in the management of depression are pharmacological and psychotherapeutic interventions. Both, however, can present challenges for geriatric patients. The remission rates after 3 trials of antidepressant treatment are around 60–70%, leaving approximately 30% of patients resistant to antidepressant treatment [6]. However, the use of antidepressants in the elderly has been guided mostly by research on younger subjects [7,8]. Whether the results from these studies are transferable to elderly patients remains questionable. As an example, a clinical trial recruiting patients over 75 years of age showed no significant efficacy of citalopram over placebo in the treatment of depression [9]. In addition, somatic comorbidities and often extensive polypharmacy may limit the choice of antidepressant medication, with common concerns including anticholinergic and cardio-circulatory side effects or sedation [2]. Furthermore, the use of psychotherapy can be limited by cognitive impairment and limited availability [8]. Electroconvulsive therapy can be very effective and even lifesaving in the case of severe and psychotic depression, but it has a reputation for severe cognitive side effects and carries the risk of anesthesia [10,11].

Non-invasive brain stimulation (NIBS) procedures, on the other hand, are less affected by such limitations and carry a small risk of side effects [12]. Repetitive transcranial magnetic stimulation (rTMS) is a safe, non-invasive neuromodulation therapy for a variety of psychiatric disorders. Study results have been most convincing for the treatment of depression [13]. rTMS as a treatment for depression is usually applied over the dorsolateral prefrontal cortex (DLPFC) and induces a magnetic field that results in the depolarization of underlying neurons and the modulation of the neural circuitry involved in emotion regulation and depressive symptoms [14,15].

While rTMS is recognized as overall safe with a low incidence of adverse events in elderly patients [16], the evidence base for its application is less valid than for younger patients due to smaller sample sizes and the heterogeneity of treatment protocols [15,17,18]. Early high-quality studies found rTMS to show more effectiveness when applied in the first year of the onset of a depressive episode and in patients below the age of 65 [10,19], leading to the assumption that rTMS is a less-than-optimal option for geriatric depression.

Large studies and resulting meta-analyses mainly included younger patients, with reported mean ages in meta-analytic syntheses ranging from 27 to 61 [17].

A theoretical difficulty in treating elderly patients that has been suggested is age-related frontodominant brain atrophy, which may limit the validity of motor threshold (MT) determination for assessment of treatment intensity because, in theory, it makes reaching the prefrontal cortex more challenging. It has been suggested that because of this, higher treatment intensities are needed in this patient population [10,17]. Individual neuronavigated identification of the DLPFC and determination of treatment intensity under consideration of the coil cortex distance has been suggested as a possible solution; however, its clinical relevance remains to be proven [13].

A recent meta-analysis concluded rTMS to be significantly superior to sham for reducing the severity of depression and for remission and response induction in patients aged 50 or older [20].

Nevertheless, evidence for the effectiveness of rTMS in the elderly, as defined as patients above the age of 60, remains scarcer and therefore weaker than for their younger counterparts. In our analysis, we aimed to directly compare the responses of different age groups with depression to rTMS treatment in a large sample.

## 2. Materials and Methods

A retrospective cohort of patients with depression who were treated with rTMS at the Center for Neuromodulation Regensburg (Germany) between 2002 and 2020 was included in the analysis. Patients gave written, informed consent to treatment. The retrospective analysis of clinical data was approved by the local ethics committee (20-2117-104) and conducted in accordance with the declaration of Helsinki. The inclusion criteria were: naïve to rTMS (in cases of repeated rTMS treatments, only the patient’s first treatment with rTMS was considered), diagnosis of depression according to ICD-10 of F31-F33, a completed Hamilton depression rating scale (HDRS) at the beginning and end of the rTMS treatment, and absence of a serious somatic illness. Both inpatients and outpatients were included. Based on these criteria, a sample of 505 patients was collected for this analysis. We have previously reported on subsets of this cohort, but with different and/or smaller samples and different objectives [21,22,23,24,25]. Treatment effects were assessed by the 21-item HDRS, which is a well-known and widely used scale in clinical and research settings that measures the severity of depression. The 21-item HDRS is a reliable depression scale considering internal consistency, inter-rater, and test-retest reliability [26,27]. Each item is rated from 0 to 2 or 0 to 4, with higher scores indicating greater severity of symptoms.

A total of 15.5% (78 out of 505) of these patients were 60 years old or older, defined as the elderly group of depressed patients. The descriptive sample characteristics are shown in Table 1 (for details, see also results). Different study protocols were used. 381 patients received 2000 pulses over the left prefrontal cortex applied at 20 Hz. A total of 75 patients also received left frontal facilitating protocols (10 Hz, 1000 pulses: *n* = 10; 10 Hz, 2000 pulses: n = 15; intermittent theta burst stimulation, 600 pulses (iTBS): n = 20; 3 times iTBS with a break of 15–20 min in between: n = 13; iTBS neuronavigated according to the border between the anterior and middle third of the middle frontal gyrus: n = 17 [22]). A total of 13 patients were treated on the medial prefrontal cortex (10 Hz, 2000 pulses), and 32 were stimulated on both the left and right DLPFC in consecutive order (13 with 1 Hz right and 10 Hz left, each side 1000 pulses; 17 with continuous TBS right and iTBS left, each side 1200 pulses). Four patients were stimulated with specific and non-standard protocols. All treatments were carried out with different machines from the MagPro series (MagVenture A/S, Farum, Denmark). Except for the medial prefrontal cortex stimulation, all treatments were carried out with a figure-of-eight coil. The coil was held stable during the treatment by a mechanic’s holding arm. The relative position of the head to the coil was adjusted if it changed during the stimulation. Resting motor thresholds were determined by the method suggested by Rossini and Rothwell, which is the threshold for which 4 out of 8 pulses elicit motor-evoked potentials above 50 µV at the hotspot of motor cortex activation [28].

All data were analyzed using SPSS (IBM Corp., Armonk, NY, USA; Version 24.0.0.0). The significance level was set at *p* < 0.05. For group comparisons, we used Student *t*-tests or chi-square tests, depending on the scales of measurement. Response was defined as a decrease in the HDRS total score of at least 50% from pre- to post-rTMS and remission as a HDRS score at the end of treatment below 11 points. As measures for effect size, we used Cohen’s d for the relative and absolute change in the HDRS total score as indicated by G*Power 3.1.9.2 [29]. As sample sizes were different, we repeated the *t*-tests non-parametrically with Mann–Whitney U tests and could replicate the findings in Table 1. To rule out the possibility that age findings are independent of arbitrarily dividing the sample into two groups, we analyzed the influence of age on relative and absolute change in depression with correlations using Pearson and Spearman coefficients.

As the sample is highly heterogeneous with respect to the treatment protocols, we repeated the analyses with a homogeneous sample of 381 patients receiving 2000 pulses at 20 Hz over the left prefrontal cortex. The results are exactly the same.

## 3. Results

Groups did not differ significantly with respect to depression type (unipolar or bipolar), depression severity, or treatment parameters, but for age (obviously) and sex (Table 1). In the group of elderly patients, a higher proportion of females was present. Overall, patients showed an amelioration of symptoms as indicated by a significant decrease in the HDRS-21 sum score (T = 20.582; df = 504; *p* < 0.001; d = 0.916, Table 1). Both groups did not differ significantly with respect to treatment efficacy, as indicated by the absolute and relative changes in the HDRS-21 sum score. The effect sizes for group contrasts were negligible. Also, response and remission rates based on the HDRS-21 sum score were not significantly different (Table 1). Correlation analyses also did not show a significant association between changes in depression and age (all r-values below 0.014).

Table 2 indicates the frequency of medication taken. A statistical analysis of the intake of tetracyclic antidepressants could not be performed due to low overall numbers. Elderly patients tended to take higher numbers of mood stabilizers (tending towards significance). Taking into account sex and intake of mood stabilizers by analysis of covariance again showed no significant group difference for the absolute (F = 0.013; df = 1.430; *p* = 0.908) and relative (F = 0.042; df = 1.430; *p* = 0.837) change of HDRS-21.

**Figure 1:**
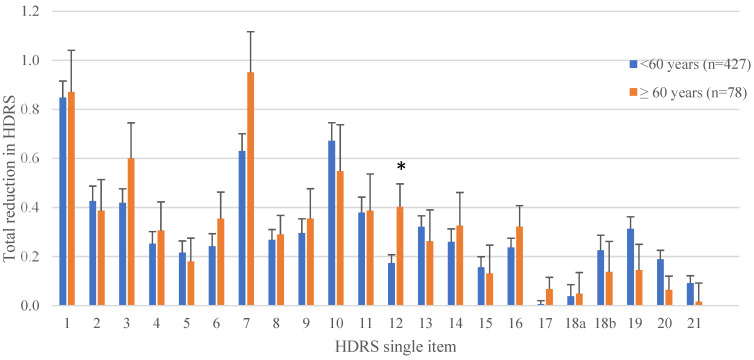
Total reduction of HDRS single-item severity after rTMS treatment. The *x*-axis represents the number of HDRS single items (see Table 3 for item descriptions). *y*-Axis represents the value of the total reduction in HDRS score for the corresponding single item. * represents a *p*-value < 0.05.

On a HDRS single item level, patients over 60 showed a significantly (*p* = 0.009) superior reduction for the item “appetite” and a superior reduction tending towards significance (*p* = 0.068) for the item “work and interests” (Figure 1 and Table 3).

## 4. Discussion

Our retrospective analysis of 505 patients showed a similar decrease in symptoms of depression for patients over 60 as well as for those below. Response and remission rates were around 30%.

Groups did not differ significantly in baseline characteristics, with the exception of a significantly higher proportion of female patients in the elderly group (65% in the older versus 52% in the younger age group). While depression is often cited to be more common in women [30], this finding might not hold true over the total life span, and a large review of transcultural international studies found older women to score higher on measures of depressive symptoms than men and to have higher rates of a diagnosis of unipolar depression in the majority of studies [31].

We lack a definite explanation for the balanced sex ratio in the younger group and female dominance in the elderly group in our sample, but we suggest it could be that men in the elderly group are more reluctant to seek psychiatric treatment for depression due to sociocultural reasons.

We currently lack a satisfactory causal explanation for the higher use of mood stabilizers in the elder group, which tends towards statistical significance. Differences in the prescription rate of mood stabilizers might suggest differences in the grade of treatment resistance to antidepressant medication and thus distort results. However, many confounding factors may be at play, contributing to the slightly higher use in the elderly patients that cannot be adequately controlled for in our study design.

On a single-item level, the group above 60 years showed a significantly superior reduction for the item concerning appetite and a superior reduction nearing statistical significance for the item concerning work and interests. This may point to an underlying biological difference in the nature of depression between older and younger patients; however, from our study design, we cannot exclude that this is a general effect of antidepressive treatment instead of a specific rTMS effect.

Taking into account the association between rTMS and centrally acting drugs, it is worth mentioning that a study conducted in 2021 concluded that the use of Lithium, Lamotrigine, and Valproic acid had no influence on the effectiveness of rTMS treatment outcomes [24]. Another study that investigated lorazepam concluded that the use of lorazepam significantly reduces the effectiveness and response rate of rTMS treatment in patients with depression [25].

Limitations of our study include the retrospective nature of the study, the heterogeneity of treatment protocols, the lack of information on the grade of treatment resistance, and the exact medication dosages. Another limitation is the presence of pharmacological and standard-of-care psychiatric and psychotherapeutic co-therapy.

However, as shown in Table 2, we can state that both groups did not differ significantly in baseline medication intake apart from mood stabilizers, and the standard care offered to patients is comparable due to the uniformity of the cohort from one center.

We also still have limited data on the very old. With a mean age of 66 years in the elderly group, our sample had a higher mean age than the meta-analyses cited in the introduction. However, our oldest patient was also only 71 years old, highlighting the need for studies concerning effectiveness and safety in even older patients. The line drawn at 60 to divide the two patient groups is, of course, rather arbitrary, and as patients grow even older and somatic comorbidities and cognitive decline increase, non-pharmacological and non-psychotherapeutic interventions might become even more important.

The main strength of our study is the very large sample size and direct comparison with the younger age group in a relatively uniform setting utilizing the same setting and treatment protocols and modalities.

Our direct comparison also indicates that the surface-based heuristics used in clinical practice to define treatment intensity (as measured by percentage of resting motor threshold) and to identify the DLPFC yield desired clinical results in elderly patients, despite theoretical concerns about brain anatomy altered by atrophy.

At least from our data, there seems to be no reason to utilize neuronavigation due to concerns for brain atrophy and the subsequent risk of incorrect coil placement with alteration of frontal anatomy, as suggested by some authors [17]. A recent prospective study has shown no difference between neuronavigated and conventional treatment, but patients were much younger, with a mean age in their forties [32]. In addition, our treatment intensities did not nearly reach the 120% of MT suggested by some authors to account for brain atrophy with increased scalp-to-cortex distance [10].

Our results are in line with the large meta-analysis by Valiengo et al., in which rTMS has shown effectiveness in older adults and no major difference in comparison with the younger group [20]. Taken together, the results and the literature support offering rTMS for depression to elderly patients, utilizing the same protocols as in younger patients.

## 5. Conclusions

Taken together, the results support the use of rTMS as an antidepressive treatment in older adults. A loss of appetite is ameliorated by antidepressive treatment in a superior manner in patients over 60 when compared to their counterparts. A further investigation would be of interest to determine whether this is an rTMS or a general antidepressive effect. Further studies are warranted, with special interest in the treatment of even older patients, as well as prospective studies of neuronavigated treatment in this patient group to examine whether effectiveness can be increased further.

## Figures and Tables

**Table 1:** Characteristics of patients with depression.

	<60 Years (n = 427)	≥60 Years (n = 78)	Statistics for Group Contrasts
age (years)	43 ± 11	66 ± 5	T = 18.532; df = 503; *p* < 0.001
sex (female/male)	222/205 (52% female)	51/27 (65% female)	χ^2^ = 4.764; df = 1; *p* = 0.029
resting motor threshold	43 ± 10	41 ± 9	T = 1.591; df = 500; *p* = 0.112
stimulation intensity	45 ± 8	45 ± 9	T = 0.557; df = 503; *p* = 0.578
number of pulses per session	1886 ± 395	1859 ± 450	T = 0.538; df = 503; *p* = 0.591
number of sessions per patient/treatment	18 ± 7	15 ± 6	T = 1.098; df = 503; *p* = 0.273
diagnosis (unipolar/bipolar)	389/38	70/8	χ^2^ = 0.147; df = 1; *p* = 0.702
HDRS-21 baseline	22 ± 7	21 ± 6	T = 0.705; df = 503; *p* = 0.481
HDRS-21 absolute change (from pre to post treatment)	7 ± 8	7 ± 7	T = 0.124; df = 503; *p* = 0.902; d = 0.016
HDRS-21 relative change (%; from pre to post treatment)	30 ± 37	32 ± 33	T = 0.445; df = 503; *p* = 0.657; d = 0.057
response rate [yes/no] (relative frequency of responders)	128/299 (30%)	26/52 (33%)	χ^2^ = 0.351; df = 1; *p* = 0.554
remission rate (yes/no)	153/274 (36%)	28/50 (36%)	χ^2^ < 0.001; df = 1; *p* = 0.991

**Table 2:** Medication intake.

	<60 Years (n = 366)	≥60 Years (n = 68)	Statistics for Group Contrasts (df = 1)
selective serotonin reuptake inhibitors	117 (32%)	23 (34%)	χ^2^ = 0.090; *p* = 0.764
serotonin-norepinephrine reuptake inhibitors	185 (51%)	37 (55%)	χ^2^ = 0.343; *p* = 0.558
tricyclic antidepressants	111 (30%)	15 (22%)	χ^2^ = 1.903; *p* = 0.168
tetracyclic antidepressants	0 (0%)	2 (3%)	not interpretable
monoamine oxidase inhibitors	11 (3%)	2 (3%)	χ^2^ = 0.001; *p* = 0.977
benzodiazepines	115 (31%)	22 (32%)	χ^2^ = 0.023; *p* = 0.879
z-drugs	41 (11%)	6 (9%)	χ^2^ = 0.336; *p* = 0.562
mood stabilizers	122 (33%)	31 (46%)	χ^2^ = 3.773; *p* = 0.052
antipsychotics	228 (62%)	48 (71%)	χ^2^ = 1.704; *p* = 0.192
other antidepressants	148 (40%)	32 (47%)	χ^2^ = 1.036; *p* = 0.309

The number in each cell indicates how many patients in the respective diagnostic group were taking medications of the indicated classification. Please note that for 71 out of 505 patients, no valid medication information was available.

**Table 3:** Total reduction of HDRS single-item level severity after rTMS treatment (see Figure 1).

Single Item	Total Reduction in Patients <60 Years (n = 427)	Total Reduction in Patients ≥60 Years (n = 78)	Statistics
1—Depressed mood	0.8	0.9	*p* = 0.839, d = 0.019
2—Feelings of guilt	0.4	0.4	*p* = 0.788, d = 0.037
3—Suicide	0.4	0.6	*p* = 0.216, d = 0.175
4—Initial insomnia	0.3	0.3	*p* = 0.660, d = 0.061
5—Insomnia during the night	0.2	0.2	*p* = 0.761, d = 0.043
6—Delayed insomnia	0.2	0.4	*p* = 0.364, d = 0.127
7—Work and interests	0.6	1.0	*p* = 0.068, d = 0.256
8—Retardation	0.3	0.3	*p* = 0.828, d = 0.030
9—Agitation	0.3	0.4	*p* = 0.676, d = 0.058
10—Psychiatric anxiety	0.7	0.5	*p* = 0.506, d = 0.093
11—Somatic anxiety	0.4	0.4	*p* = 0.963, d = 0.007
12—Appetite	0.2	0.4	*p* = 0.009, d = 0.365
13—General somatic symptoms	0.3	0.3	*p* = 0.608, d = 0.072
14—Genital symptoms	0.3	0.3	*p* = 0.640, d = 0.072
15—Hypochondriasis	0.2	0.1	*p* = 0.813, d = 0.033
16—Weight loss	0.2	0.3	*p* = 0.357, d = 0.128
17—Illness insight	0	0.1	*p* = 0.104, d = 0.232
18a—Diurnal variation “when”	0	0	*p* = 0.924, d = 0.013
18b—Diurnal variation “severity”	0.2	0.1	*p* = 0.553, d = 0.085
19—Depersonalization	0.3	0.1	*p* = 0.160, d = 0.196
20—Paranoid symptoms	0.2	0.1	*p* = 0.147, d = 0.202
21—Obsessive symptoms	0.1	0	*p* = 0.312, d = 0.142

## Data Availability

The datasets for this manuscript are not publicly available because the approval from the ethic committee of university of Regensburg is restricted to publication of the data results—not the datasets itself—in the corresponding journals. Requests to access the datasets should be directed to: Martin Schecklmann, Email: martin.schecklmann@medbo.de.

## References

[1] Borges M.K., Romanini C.V., Lima N.A., Petrella M., da Costa D.L., An V.N., Aguirre B.N., Galdeano J.R., Fernandes I.C., Cecato J.F. (2021). Longitudinal Association between Late-Life Depression (LLD) and Frailty: Findings from a Prospective Cohort Study (MiMiCS-FRAIL). J. Nutr. Health Aging.

[2] Avasthi A., Grover S. (2018). Clinical practice guidelines for management of depression in elderly. Indian J. Psychiatry.

[3] Luppa M., Sikorski C., Motzek T., Konnopka A., Konig H.H., G Riedel-Heller S. (2012). Health Service Utilization and Costs of Depressive Symptoms in Late Life—A Systematic Review. Curr. Pharm. Des..

[4] Hegeman J.M., Kok R.M., van der Mast R.C., Giltay E.J. (2012). Phenomenology of depression in older compared with younger adults: Meta-analysis. Br. J. Psychiatry.

[5] Szymkowicz S.M., Gerlach A.R., Homiack D., Taylor W.D. (2023). Biological factors influencing depression in later life: Role of aging processes and treatment implications. Transl. Psychiatry.

[6] Valiengo L., Pinto B.S., Marinho K.A., Santos L.A., Tort L.C., Benatti R.G., Teixeira B.B., Miranda C.S., Cardeal H.B., Suen P.J.C. (2022). Treatment of depression in the elderly with repetitive transcranial magnetic stimulation using theta-burst stimulation: Study protocol or a randomized, double-blind, controlled trial. Front. Hum. Neurosci..

[7] Giron M.S.T., Fastbom J., Winblad B. (2005). Clinical trials of potential antidepressants: To what extent are the elderly represented: A review. Int. J. Geriatr. Psychiatry.

[8] Taylor W.D. (2014). Depression in the Elderly. N. Engl. J. Med..

[9] Roose S.P., Rutherford B.R., Nelson J.C., Delucchi K.L., Schneider L.S., Alexopoulos G.S., Raskin J., Wiltse C.G., Siegal A., Sheikh J. (2004). Antidepressant Pharmacotherapy in the Treatment of Depression in the Very Old: A Randomized, Placebo-Controlled Trial. Am. J. Psychiatry.

[10] Blumberger D.M., Hsu J.H., Daskalakis Z.J. (2015). A Review of Brain Stimulation Treatments for Late-Life Depression. Curr. Treat. Options Psychiatry.

[11] McDonald W.M. (2016). Neuromodulation Treatments for Geriatric Mood and Cognitive Disorders. Am. J. Geriatr. Psychiatry.

[12] Rossi S., Antal A., Bestmann S., Bikson M., Brewer C., Brockmöller J., Carpenter L.L., Cincotta M., Chen R., Daskalakis J.D. (2021). Safety and recommendations for TMS use in healthy subjects and patient populations, with updates on training, ethical and regulatory issues: Expert Guidelines. Clin. Neurophysiol..

[13] Lefaucheur J.P., Aleman A., Baeken C., Benninger D.H., Brunelin J., Di Lazzaro V., Filipovic S.R., Grefkes C., Hasan A., Hummel F.C. (2020). Evidence-based guidelines on the therapeutic use of repetitive transcranial magnetic stimulation (rTMS): An update (2014–2018). Clin. Neurophysiol..

[14] Klomjai W., Katz R., Lackmy-Vallée A. (2015). Basic principles of transcranial magnetic stimulation (TMS) and repetitive TMS (rTMS). Ann. Phys. Rehabil. Med..

[15] Hebel T., Grozinger M., Landgrebe M., Padberg F., Schecklmann M., Schlaepfer T., Schonfeldt-Lecuona C., Ullrich H., Zwanzger P., Langguth B. (2022). Evidence and expert consensus based German guidelines for the use of repetitive transcranial magnetic stimulation in depression. World J. Biol. Psychiatry.

[16] Overvliet G.M., Jansen R.A.C., van Balkom A.J.L.M., van Campen D.C., Oudega M.L., van der Werf Y.D., van Exel E., Heuvel O.A.V.D., Dols A. (2020). Adverse events of repetitive transcranial magnetic stimulation in older adults with depression, a systematic review of the literature. Int. J. Geriatr. Psychiatry.

[17] Sabesan P., Lankappa S., Khalifa N., Krishnan V., Gandhi R., Palaniyappan L. (2015). Transcranial magnetic stimulation for geriatric depression: Promises and pitfalls. World J. Psychiatry.

[18] Iriarte I.G., George M.S. (2018). Transcranial Magnetic Stimulation (TMS) in the Elderly. Curr. Psychiatry Rep..

[19] George M.S., Lisanby S.H., Avery D., McDonald W.M., Durkalski V., Pavlicova M., Anderson B., Nahas Z., Bulow P., Zarkowski P. (2010). Daily left prefrontal transcranial magnetic stimulation therapy for major depressive disorder: A sham-controlled randomized trial. Arch. Gen. Psychiatry.

[20] Valiengo L., Maia A., Cotovio G., Gordon P.C., Brunoni A.R., Forlenza O.V., Oliveira-Maia A.J. (2022). Repetitive Transcranial Magnetic Stimulation for Major Depressive Disorder in Older Adults: Systematic Review and Meta-analysis. J. Gerontol. Ser. A.

[21] Abdelnaim M.A., Langguth B., Deppe M., Mohonko A., Kreuzer P.M., Poeppl T.B., Hebel T., Schecklmann M. (2019). Anti-Suicidal Efficacy of Repetitive Transcranial Magnetic Stimulation in Depressive Patients: A Retrospective Analysis of a Large Sample. Front. Psychiatry.

[22] Frank E., Eichhammer P., Burger J., Zowe M., Landgrebe M., Hajak G., Langguth B. (2011). Transcranial magnetic stimulation for the treatment of depression: Feasibility and results under naturalistic conditions: A retrospective analysis. Eur. Arch. Psychiatry Clin. Neurosci..

[23] Hebel T., Abdelnaim M., Deppe M., Langguth B., Schecklmann M. (2020). Attenuation of antidepressive effects of transcranial magnetic stimulation in patients whose medication includes drugs for psychosis. J. Psychopharmacol..

[24] Hebel T., Abdelnaim M.A., Deppe M., Kreuzer P.M., Mohonko A., Poeppl T.B., Rupprecht R., Langguth B., Schecklmann M. (2021). Antidepressant effect of repetitive transcranial magnetic stimulation is not impaired by intake of lithium or antiepileptic drugs. Eur. Arch. Psychiatry Clin. Neurosci..

[25] Deppe M., Abdelnaim M., Hebel T., Kreuzer P.M., Poeppl T.B., Langguth B., Schecklmann M. (2021). Concomitant lorazepam use and antidepressive efficacy of repetitive transcranial magnetic stimulation in a naturalistic setting. Eur. Arch. Psychiatry Clin. Neurosci..

[26] Hamilton M. (1960). A rating scale for depression. J. Neurol. Neurosurg. Psychiatry.

[27] Trajković G., Starčević V., Latas M., Leštarević M., Ille T., Bukumirić Z., Marinković J. (2011). Reliability of the Hamilton Rating Scale for Depression: A meta-analysis over a period of 49 years. Psychiatry Res..

[28] Kallioniemi E., Julkunen P. (2016). Alternative Stimulation Intensities for Mapping Cortical Motor Area with Navigated TMS. Brain Topogr..

[29] Faul F., Erdfelder E., Buchner A., Lang A.-G. (2009). Statistical power analyses using G*Power 3.1: Tests for correlation and regression analyses. Behav. Res. Methods.

[30] Kessler R.C. (2003). Epidemiology of women and depression. J. Affect. Disord..

[31] Girgus J.S., Yang K., Ferri C.V. (2017). The Gender Difference in Depression: Are Elderly Women at Greater Risk for Depression Than Elderly Men?. Geriatrics.

[32] Hebel T., Göllnitz A., Schoisswohl S., Weber F.C., Abdelnaim M., Wetter T.C., Rupprecht R., Langguth B., Schecklmann M. (2021). A direct comparison of neuronavigated and non-neuronavigated intermittent theta burst stimulation in the treatment of depression. Brain Stimul..

